# Detection of *Histoplasma capsulatum* in Bats from the Brazilian Western Amazon

**DOI:** 10.3390/jof11040314

**Published:** 2025-04-16

**Authors:** Jhonatan Henrique Lima da Rocha, Tamyres Izarelly Barbosa da Silva, Rair de Sousa Verde, Guilherme Henrique Reckziegel, Cíntia Daudt, Daniel Archimedes da Matta, Francisco Glauco de Araújo Santos

**Affiliations:** 1 Center for Biological and Nature Sciences, Federal University of Acre, Rio Branco 69920-900, Brazil; tamyres.silva@ufac.br (T.I.B.d.S.); rair.verde@gmail.com (R.d.S.V.); guilherme.reckziegel@sou.ufac.br (G.H.R.); cintia.daudt@ufac.br (C.D.); francisco.araujo@ufac.br (F.G.d.A.S.); 2 Rodolphe Mérieux Laboratory, Charles Mérieux Center for Infectious Diseases, FUNDHACRE, Rio Branco 69920-193, Brazil; darchimedes@hotmail.com

**Keywords:** chiroptera, fungal pathogen, One Health, reservoirs, Zoonoses

## Abstract

*Histoplasma capsulatum* is a saprophytic dimorphic fungus that causes histoplasmosis, a systemic infectious disease of relevance to public health. Bats can be important agents in the epidemiological cycle of the disease since they act as reservoirs of microorganisms. The aim of this study was to detect *Histoplasma capsulatum* in the lung tissue of bats captured in urban forest fragments in the municipality of Rio Branco, Acre, in the Western Amazon. Twenty-two bat species were captured from five urban forest fragments. The samples taken were subjected to histopathological, mycological, and molecular analysis. Among the 96 animals analyzed, the fungus was detected in 32.29% (31/96). This was the first study to detect the pathogen in bats in the Western Amazon. It is also the first record of the fungus being detected in six bat species. The state of Acre is located in a region with a rich diversity of bats. Furthermore, this area is constantly suffering from climatic and environmental changes that can favor the emergence and re-emergence of diseases. Thus, active epidemiological research and surveillance of neglected fungal infections are essential, especially considering the concept of One Health.

## 1. Introduction

*Histoplasma capsulatum* is a dimorphic and saprophytic fungus with a cosmopolitan distribution that is potentially pathogenic to humans and animals. This microorganism is responsible for causing a widespread mycotic zoonosis called histoplasmosis [1,2]. The designation *H. capsulatum* corresponds to the asexual phase, while in its telemorphic phase, it receives the nomenclature *Ajellomyces capsulatus* [3].

Until recently, *H. capsulatum* was considered to be the only species in the genus with three varieties: var. *capsulatum*, var. *duboisii*, and var. *farciminosum.* However, molecular studies have determined the existence of new genetically distinct groups made up of eight clades (North American class 1 clade; North American class 2 clade; Latin American group A clade; Latin American group B clade; Australian clade; Dutch clade; Eurasian clade; and African clade). Thus, the genus *Histoplasma* came to be considered a complex of cryptic species, in which *H. capsulatum* var. *capsulatum* was found as a common ancestor in all eight clades [4,5,6].

*H. capsulatum* is categorized as a high-priority fungal pathogen according to the World Health Organization [7]. In Brazil, histoplasmosis is reported to be endemic, especially in the Midwest, Southeast, and South regions. Among the 26 Brazilian states, 19 have already reported cases of human histoplasmosis [8].

Bats are extremely important in the epidemiological cycle of histoplasmosis since they can act as reservoirs and contribute to the dispersal of the fungus in the environment through their feces [9]. The isolation of *H. capsulatum* in bats has been described in five Brazilian states [8]. In the Amazon biome, only the state of Pará has data on the detection of the pathogen in bats [10].

Considering the importance of this microorganism to our health and the role of bats in dispersing this pathogen, as well as the lack of eco-epidemiological data in Brazil, especially in the Amazon region, this study aimed to detect *H. capsulatum* in bats caught in urban forest fragments in Rio Branco, Acre, in the Western Amazon.

## 2. Materials and Methods

### 2.1. Ethical Principles

This study was filed with the Biodiversity Authorization and Information System (SISBIO) under registration number 83196-2 and authorized by the Animal Use Ethics Committee (CEUA) of the Federal University of Acre (UFAC) under license number 31/2022 on 24 December 2022.

### 2.2. Study Area and Capture and Identification of Bats

**Figure 1:**
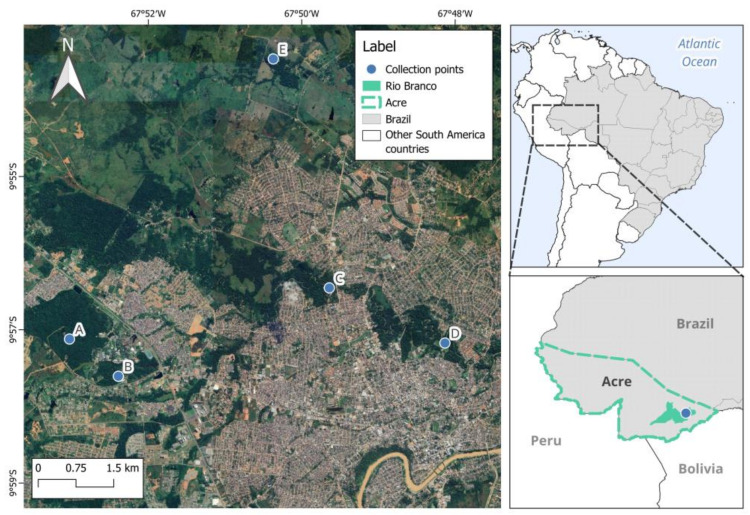
Chiropteran capture points in forest fragments in the municipality of Rio Branco, Acre.

The bats were captured between December 2022 and October 2023 in five urban forest fragments in the municipality of Rio Branco, Acre (Figure 1).

The animals were captured using seven mist nets, each measuring 12 m × 2.5 m, set up after sunset (6:00 p.m.) and left open for 6 h; they were monitored every 30 min. The nets were placed on transects at the edges of the forest fragments and near water sources.

After capture, the animals were removed from the net and placed in cotton bags for an assessment of their biometrics, in which weight, forearm length, wingspan length, sex, and age were recorded [11]. The species were identified according to their morphological and morphometric characteristics based on taxonomic identification keys [12,13].

### 2.3. Collection of Biological Samples

The animals were anesthetized with ketamine hydrochloride (Vetanarcol, Laboratórios König, Brazil), at a dose of 50 mg/kg intramuscularly, in the pectoral muscle, fast-acting, and euthanized through exsanguination by cardiac puncture, in accordance with the recommended bioethical procedures [14,15].

To collect the lungs, the euthanized animals were placed in a class II biological safety laminar flow cabinet. After antisepsis of the animal’s thoracic region with 70% alcohol, thoracotomy was performed, with mechanical extraction of the sternum and ribs to remove the organs [16]. The lungs were fractionated into equivalent portions and stored in sterile tubes for laboratory processing.

### 2.4. Histopathological Diagnosis

The collected lung fractions were fixed in 10% formaldehyde for 24 h. After being fixed, the tissue samples were subjected to standard histopathological processing in an automated histotechnician. The histopathological slides were stained with hematoxylin and eosin (HE) to visualize possible morphological alterations, as well as subjected to the special Grocott–Gomori staining process (GMS) to highlight fungal structures [17].

### 2.5. Microbiological Culture

For the fungal culture, the bat lung fragments were macerated and placed in sterile tubes containing 3 mL of saline solution with chloramphenicol 200 mg/L and homogenized in a vortex for 60 s. The final solution was distributed at a rate of 0.5 mL/tube in three culture media: Sabouraud dextrose agar (SDA), brain–heart infusion agar (BHI), and Mueller Hinton Broth with L-cysteine and hen egg yolk (ML–egg yolk). As this is a dimorphic fungus, the cultures were incubated at two temperatures to increase the chance of isolation, so three tubes of each medium were incubated at 25 °C, and the other three were incubated at 37 °C, for a period of up to 40 days [16].

The isolates were identified based on the morphology of the colonies after growth in the culture media. For macroscopic characteristics, color, texture, surface, and growth time were taken into account. Regarding the microscopic characteristics, microcultures of suspected colonies were created. The slides were stained with lactophenol blue to better visualize the structures, and they were evaluated using optical microscopy [18].

To confirm the suspected isolates, reversion to the yeast phase was carried out by sowing the fungus in the mycelial phase in ML–egg yolk and BHI media incubated at 37 °C for approximately 7 days [16]. Only *Histoplasma* species isolates were used in this study.

### 2.6. DNA Extraction

The genomic DNA of the lung fragment samples was extracted using the QuatroG Biotecnologia Kit (bacteria, tissue and blood genomic DNA extraction kit, Porto Alegre, RS, Brazil) according to the manufacturer’s recommendations.

To detect the pathogen, a fragment of the *Hcp100* gene, which encodes a 100kDA protein from *H. capsulatum*, was amplified using the Nested PCR method, as proposed by Bialek et al. (2002) [19] with modifications. The set of primers used consisted of HcI (5′GCGTTC-CGA-GCC-TTC-CAC-CTC-AAC-3′) and HcII (5′-ATGTCC-CAT-CGGCG-CCG-TGT-AGT-3′) as external primers and Hc III (5′-GAG- ATC-TAG-TCG-CGG-CCA-GGT-TCA-3′) and Hc IV (5′-AGGAGA-GAA-CTG-TAT-CGG-TGG-CTT-G-3′) as a set of internal primers resulting in the amplification of a final product of 210 bp.

The two PCR steps contained 5 µL of total DNA (in the first step) and 2 µL of the amplicon (in the second step), plus 10 nM of Tris-HCL, pH 8.3, 1.0 mM of MgCl2, 0.2 mM of each dNTP (nested 50 uM), 10 pmol of each primer, and 1.5 U of *Taq* polymerase, adjusted to a final volume of 25 µL.

The first PCR reaction consisted of an initial denaturation step at 94 °C for 5 min followed by 35 cycles of 94 °C for 30 s, 50 °C for 30 s, and 72 °C for 1 min. The final extension was at 72 °C for 5 min. The protocol for the second reaction was an initial denaturation step at 94 °C for 5 min followed by 35 cycles of 94 °C for 30 s, 63 °C for 30 s, and 72 °C for 1 min with a final extension at 72 °C for 5 min [10].

DNA from *H. capsulatum*, clinical strain G217B (ATCC; Rockville, MD, USA) (ATCC 26032), provided by Medical Mycology Laboratory of the Oswaldo Cruz Foundation Fiocruz, Rio de Janeiro, Brazil, was used as a positive control. Deionized ultrapure water was used as a negative control. The reaction products were electrophoresed with SYBR safe stain (Thermo Fisher Scientific, Waltham, MA, USA) on agarose gel (2%) in TAE buffer. The amplified products were estimated using a standard 50 bp ladder (Ludwig Biotecnologia Ltd.a, Bela Vista, São Paulo, Brazil). Visualization was performed on a transilluminator under UV light and photodocumented.

### 2.7. Sequencing and Phylogenetic Analysis

Positive samples were purified using the PureLink^®^ Quick PCR Purification Kit (Invitrogen, Carlsbad, CA, USA) following the manufacturer’s recommendations. Sequencing was carried out using the BigDye Terminator v.3.1 Cycle Sequencing Kit (Applied Biosystems, Waltham, MA, USA) using the AB 3500 Genetic Analyzer (Applied Biosystems, Waltham, MA, USA).

The sequences were trimmed and edited using the Geneious version 2025.0.2 software and compared with the online database (GenBank) using the BLASTn tool (https://blast.ncbi.nlm.nih.gov/Blast.cgi, accessed on 14 December 2024) [20]. The sequences with the greatest similarity to the sequences obtained in this study were collected, as well as reference sequences deposited in the online database.

Subsequently, the alignment was carried out using the Clustal W multiple sequence alignment program [21], and the evolutionary history was inferred using the Maximum Likelihood method and the Kimura 2-parameter model, according to which was the best model tool [22], using the MEGA X software version 10.2.6 [23]. The reliability of the generated tree was tested with 1000 replicates, and bootstrap values > 50% were considered significant.

## 3. Results

At points A, C, D, and E, 20 bats were captured per region, while at B, 16 chiropterans were captured, totaling 96 animals, in which *H. capsulatum* was detected in 32.29% (31/96). The family, species, sex, age group, capture point, and diagnostic test used to infer the occurrence of the fungus in each animal can be seen in Table 1.

**Figure 2:**
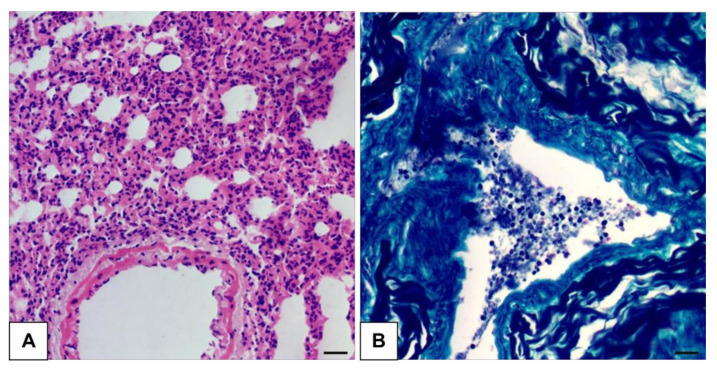
Photomicrographs of the lung parenchyma of a bat infected with *H. capsulatum*. (**A**) Thickened alveolar septa, with the presence of a chronic lymphomonocytic inflammatory infiltrate, characteristic of diffuse interstitial pneumonia. 400× magnification, HE (BAR = 10 μm). (**B**) Oval, agglomerated yeasts of varying sizes, some with budding, showing morphology compatible with *Histoplasma* spp. 400× magnification, GMS (BAR = 10 μm).

Among the positive animals, the histopathological examination revealed alterations consistent with diffuse interstitial pneumonia in 45.16% of these animals (14/31) (Figure 2A); however, as this is a common alteration in various respiratory disorders [24], it cannot be inferred that the cause is due to fungal infection. In relation to the special staining to visualize fungal structures, 61.29% (19/31) of the animals had morphologies characteristic of *Histoplasma* spp. (Figure 2B).

**Figure 3:**
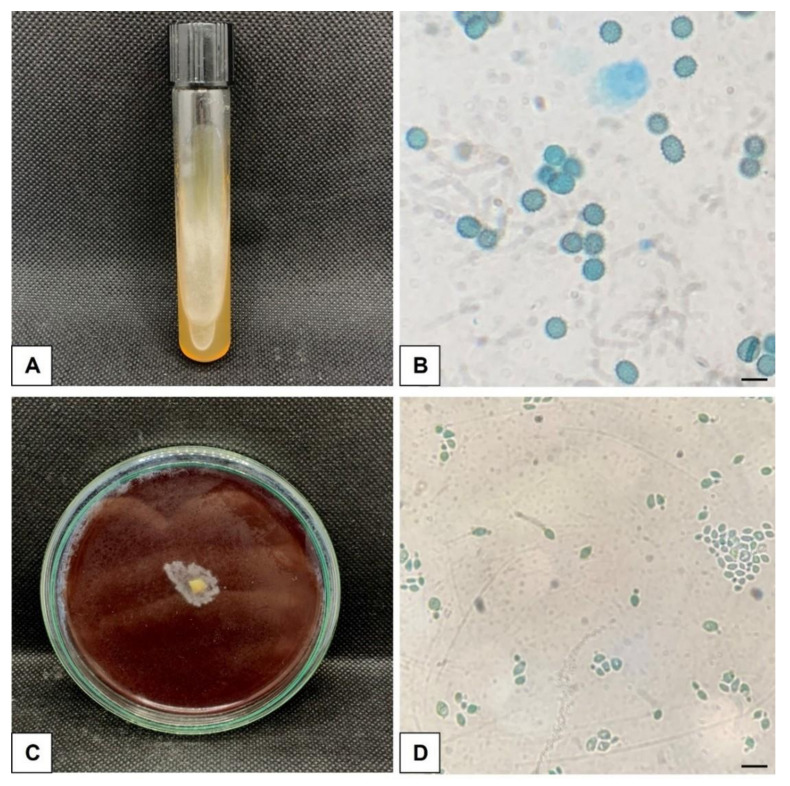
Morphology of *H. capsulatum* in mycological culture. (**A**) Mycelial phase of *H. capsulatum* in DSA. (**B**) Microscopic aspects of the mycelial phase with characteristic macroconidia. 1000× magnification, lactophenol cotton blue staining (BAR = 10 μm). (**C**) Cultivation of the yeast phase in BHI medium enriched with blood. (**D**) Microscopic aspects of the yeast phase with single budding yeasts. Magnification 1000×, lactophenol cotton blue staining (BAR = 10 μm).

In the microbiological examination, an adult male *Artibeus lituratus* captured at Point A presented colonies with macro (Figure 3A) and micro (Figure 3B) morphological characteristics suggestive of *H. capsulatum* in its mycelial form. The detection of the fungus was confirmed by the in vitro reversion test, in which the dimorphism from the filamentous to the yeast-like phase was observed (Figure 3C) and characteristic yeasts were observed under microscopy (Figure 3D).

**Figure 4:**
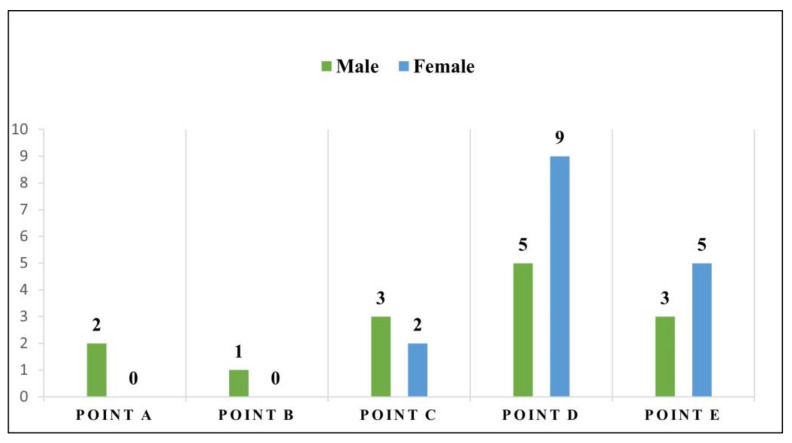
Distribution of Histoplasma capsulatum by sex of animal and catch area in Rio Branco, Acre.

In the molecular analysis of the lung fragments submitted to Nested PCR, 31.25% (30/96) of the samples were detectable for *H. capsulatum*. Among the 30 positive animals, 53.33% (16/30) were females, while 46.67% (14/30) were males. Among the capture areas, Point D had the highest frequency of detectable animals with 46.67% (14/30), followed by Point E with 26.67% (8/30), Point C with 16.67% (5/30), Point A with 6.67% (2/30), and Point B with 3.33% (1/30) (Figure 4).

**Figure 5:**
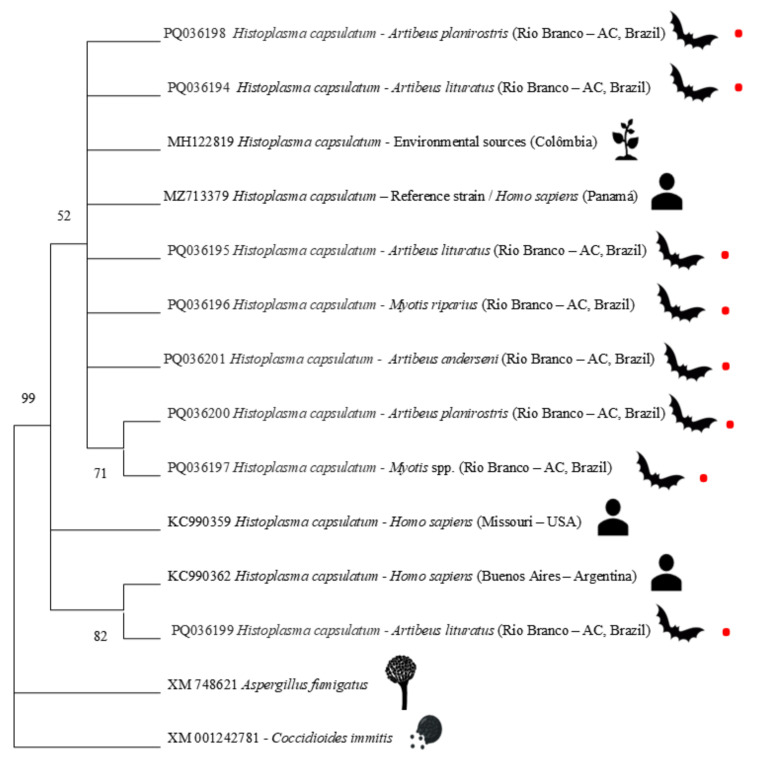
Phylogenetic tree based on the sequences of partial 100 kDa protein genes (*Hcp100* gene) of *Histoplasma capsulatum* detected in bats from the Western Amazon. This analysis involved 14 sequences: 8 from bats in the Western Amazon (marked with red dots), 4 representing isolates different detection sources, as well as non-*Histoplasma* sequences. The bootstrap percentage of trees in which the associated taxa grouped together is shown next to the branches.

Ten PCR-positive samples were selected for sequencing and submitted to BLASTx. Of these, eight showed a high percentage of identity (96 to 100%) with the partial sequences of the *H. capsulatum* 100 kDa protein (*Hcp100* gene). Two sequenced samples could not be analyzed due to poor sequencing quality. The phylogenetic analysis of the samples obtained in this study compared to the most similar sequences and reference sequences obtained from GenBank can be seen in Figure 5. All the sequences obtained in this study have been deposited in the online database under the following access numbers: PQ036194, PQ036195, PQ036196, PQ036197, PQ036198, PQ036199, PQ036200, and PQ036201.

## 4. Discussion

Studies on the detection of *H. capsulatum* in bats in the Amazon region are still scarce. To date, only Silva et al. [10] have carried out this type of research, specifically on animals captured in the state of Pará in the Eastern Amazon. As far as we know, our work is the first to provide data on the fungus in bat lungs in the Western Amazon.

In our study, *H. capsulatum* was detected in 11 species of bats, including the first record of the fungus being detected in 6 species of chiropets, namely *Artibeus anderseni*, *Artibeus cinereus*, *Artibeus planirostris*, *Hsunycteris thomasi*, *Myotis riparius*, and *Rhynchonycteris naso.* Gugnani and Denning [25] recorded 48 bat species in which *H. capsulatum* was isolated directly from animal tissue samples from 1962 to 2021. Our results contribute to epidemiological knowledge and broadens the understanding of the role of these animals in the ecoepidemiology of *H. capsulatum*.

Our findings indicate that not all bats infected with *Histoplasma* spp. show lesions characteristic of histoplasmosis, although the pathogen is detectable in other tests. This result suggests a possible resistance of the bats to the infectious process caused by the fungus. Previous studies, such as those by Taylor et al. [26] and Suárez-Álvarez et al. [27], also observed through histological analysis of naturally and experimentally infected bats that these animals exhibited minimal inflammatory reactions. This reinforces the hypothesis of resistance, although the pathogenesis of *Histoplasma* spp. varies according to factors specific to the pathogen and the host [28].

Among the factors that contribute to the tolerance of bats to the pathogen is the variation in the body temperature of these animals during torpor (around 10 °C) and flight (average of 40 °C), which can interfere with the dimorphism of the fungus and reduce its infectivity [25,29,30]. In addition, the characteristics of the immune system of these animals seem to favor a chronic and asymptomatic infection, allowing them to eliminate viable yeasts in the feces through the intestinal villi, which reinforces their role as natural reservoirs of the fungus [31,32,33].

In the present study, no research was carried out on the immune response of bats to *H. capsulatum*. However, T-cell lymphocyte culture reactions from bats demonstrated delays when compared to other mammals, assuming that these animals are capable of truncating an immunological response to mitigate the immunopathology promoted by the pathogen [32].

Histoplasmosis can be diagnosed using presumptive methods such as histopathology with special staining and confirmatory methods such as mycological isolation or PCR [2,34]. In our study, microbiological isolation, although the gold standard, was less sensitive than PCR. This was possibly due to the low load of the microorganism in the lung tissue of bats, which are natural reservoirs. In one sample, the only fungal isolate was identified in culture and histopathology but not in PCR, a result like that of Paz et al. [35], who attributed the discrepancy to factors such as insufficient DNA extraction or technical limitations [36]. These findings reinforce the need for further studies to overcome these diagnostic difficulties.

In addition to being a faster detection method, PCR offers greater laboratory safety and allows for evolutionary and genetic diversity analyses, contributing to the molecular epidemiology of the microorganism [37,38]. In our study, this method showed the highest sensitivity for detection in bats, with 31.25% of samples (30/96) being positive, a result like that of Salomão et al. [39] and Santos et al. [40] who obtained 25.3% (249 samples) and 34.8% (89 samples) positivity, respectively, when analyzing lung tissue. Studies using organ pools, such as Dias et al. [16], Paz et al. [35], and Silva et al. [10], showed lower frequencies of 3.6% (87/2427), 8.1% (14/172), and 2% (2/100) positivity, highlighting the greater efficiency of PCR in molecular diagnosis in asymptomatic reservoirs.

The phylogeny of the samples detected in this study formed significant clades, revealing phylogenetic proximity to isolates of human and environmental origin and pointing to the existence of a regional zoonotic cycle. Muniz et al. [41] carried out the first molecular typing study of the pathogen in Brazil, in which samples of different origins (human, animal, and environmental) obtained in the state of Rio de Janeiro showed high genetic similarity, suggesting that an environmental niche acts as a possible source of infection for individuals in the region.

In terms of overall reliability (*bootstrap*), the internal relationships show variability, forming clusters with supports of 52, 71, and 82 between the bat sequences and other reference sequences. This variation in confidence values was also observed in other studies such as those by González-González et al. [42] and Moreira et al. [43]. It is suggested that these distinctions in the phylogenetic groupings may be related to the possible genetic diversity of the *H. capsulatum* detected in the chiropters of this region, possibly due to ecological and geographical issues.

Regarding cases of invasive diseases in humans caused by *H. capsulatum* in Brazil, the real incidence is unknown since mycotic diseases are not of mandatory notification [44]. Among the 26 Brazilian states, 19 have already reported human histoplasmosis cases; however, no data are recorded for the state of Acre [8]. Therefore, it points to a scenario where there is diagnostic failure and serious disease underreporting, as we verified high positivity rates of *H. capsulatum* in bats captured in urban areas, demonstrating the presence of the pathogen in the region.

In a climate change scenario, fungal infections can be boosted, resulting in an increase in the incidence of cases such as histoplasmosis and other invasive fungal infections. These climate changes can also affect the attributes of pathogens and their interactions with the environment and hosts, creating favorable conditions for the emergence of new adapted or multidrug-resistant strains [45,46]. In parallel, urbanization processes have promoted a decline in the population of several species of wild animals. However, bats, especially generalist species, show remarkable resilience and adaptability to anthropized environments by using urban forest fragments or human constructions as shelters [47]. The presence of these animals in urban centers can create favorable conditions for the proliferation of fungi, generating new focal points of infection [48,49,50].

## 5. Conclusions

The high rate of detection of *H. capsulatum* in bats in this study places the state of Acre, especially Rio Branco, as a place of great relevance to the occurrence of the pathogen in the Amazon rainforest biome. It emphasizes the importance of research and the active epidemiological surveillance of neglected fungal infections, especially considering the concept of One Health.

## Figures and Tables

**Table 1:** Detection of *H. capsulatum* in the lungs of bats caught in urban forest fragments in the municipality of Rio Branco, Acre.

Family	Taxon	Sex	Age Range	Collection Points	Change in Lung Parenchyma?	Suggestive of *Histoplasma* spp. in GMS	Fungal Culture	Nested PCR
Phyllostomidae	*Artibeus anderseni*	M	Adult	E	Yes	No	−	**+**
	*Artibeus anderseni*	F	Adult	E	Yes	Yes	−	**+**
	*Artibeus lituratus*	M	Adult	A	No	Yes	+	**−**
	*Artibeus lituratus*	M	Adult	A	No	Yes	−	**+**
	*Artibeus lituratus*	M	Adult	B	Yes	Yes	−	**+**
	*Artibeus lituratus*	M	Adult	C	Yes	Yes	−	**+**
	*Artibeus lituratus*	F	Adult	C	Yes	No	−	**+**
	*Artibeus lituratus*	F	Adult	D	Yes	No	X	**+**
	*Artibeus lituratus*	M	Adult	D	No	Yes	X	**+**
	*Artibeus lituratus*	F	Adult	D	No	No	X	**+**
	*Artibeus lituratus*	M	Adult	D	No	Yes	X	**+**
	*Artibeus lituratus*	F	Adult	D	No	Yes	X	**+**
	*Artibeus lituratus*	M	Adult	D	Yes	Yes	X	**+**
	*Artibeus planirostis*	F	Adult	E	Yes	Yes	−	**+**
	*Artibeus planirostis*	F	Adult	C	No	Yes	−	**+**
	*Artibeus cinereus*	F	Adult	D	No	Yes	X	**+**
	*Carollia perspicillata*	F	Adult	D	No	Yes	X	**+**
	*Carollia perspicillata*	F	Adult	D	No	Yes	X	**+**
	*Glossophaga soricina*	F	Adult	D	No	No	X	**+**
	*Glossophaga soricina*	F	Adult	D	No	Yes	X	**+**
	*Hsunycteris thomasi*	M	Adult	D	Yes	Yes	X	**+**
	*Phyllostomus hastatus*	F	Adult	E	Yes	No	X	**+**
	*Phyllostomus hastatus*	M	Adult	C	No	No	−	**+**
Vespertilionidae	*Myotis riparius*	M	Adult	A	No	Yes	−	**+**
	*Myotis riparius*	F	Adult	E	No	Yes	−	**+**
	*Myotis riparius*	M	Adult	D	Yes	No	−	**+**
	*Myotis riparius*	F	Adult	D	No	No	−	**+**
	*Myotis* sp.	M	Adult	C	Yes	No	−	**+**
Emballonuridae	*Rhynchonycteris naso*	M	Adult	E	No	Yes	X	**+**
	*Rhynchonycteris naso*	M	Adult	E	Yes	No	X	**+**
	*Rhynchonycteris naso*	F	Adult	E	Yes	No	X	**+**

Caption: F = female; M = male; + = positive; − = negative; X = unrealized.

## Data Availability

All data generated or analyzed during this study are included in this published article.
